# Supplementary material for: Short-Term Estivation and Hibernation Induce Changes in the Blood and Circulating Hemocytes of the Apple Snail Pomacea canaliculata
Source: Metabolites. 2023 Feb 16;13(2):289. doi: 10.3390/metabo13020289 (PMC9963190; doi:10.3390/metabo13020289)

metabolites

Article

Short-term estivation and hibernation induce changes in the blood and circulating hemocytes of the apple snail *Pomacea canaliculata*

Supplementary material.

Figure S1: DCF fluorescence in total circulating hemocytes. The upper histograms of DCF fluorescence were used to classify two subpopulations with different DCF intensity. Only the DCF^{+} population was used for further statistical analysis. The lower histograms show examples of percent inhibition by CCCP. The statistical analysis is presented in Fig. 4 of the manuscript.

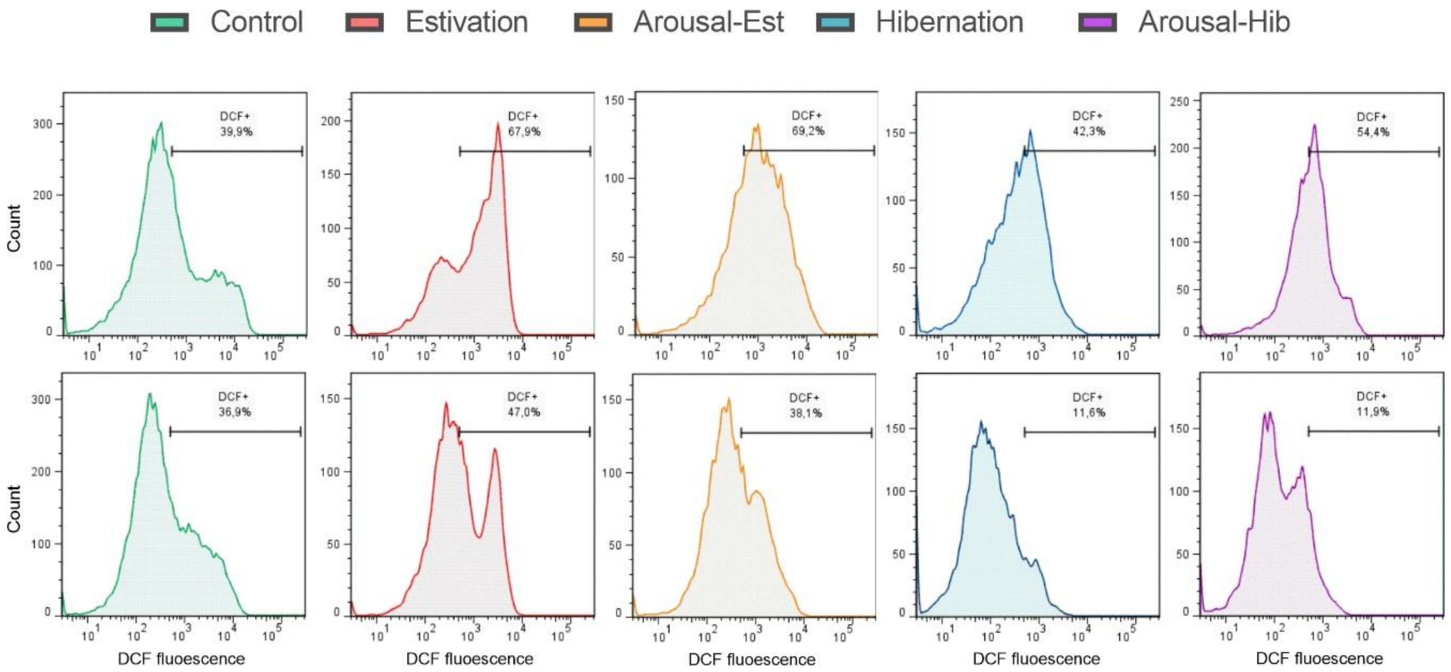

Supplement: Supplementary file 1 [file metabolites-13-00289-s001.zip › metabolites-2178057-supplementary.pdf]
